# Joint association of physical activity and screen exposure with subsequent suspected developmental coordination disorder in preschool children: a nationwide population-based study

**DOI:** 10.1186/s12966-026-01943-8

**Published:** 2026-07-06

**Authors:** Liang Du, Xiao Wang, Jing Li, Winnie Wan-Yee Tso, Chai Yin Charlie Fan, Gareth J. Williams, Wenchong Du, Jing Hua

**Affiliations:** 1 Department of Women’s and Children’s Health Care, Shanghai Key Laboratory of Maternal Fetal Medicine, Shanghai First Maternity and Infant Hospital, School of Medicine, Tongji University, Shanghai, 201204 China; 2 Jiaxing Maternity and Child Health Care Hospital, Jiaxing, China; 3 Department of Paediatrics and Adolescent Medicine, Hong Kong Children’s Hospital, Hong Kong SAR, China; 4 Department of Paediatrics and Adolescent Medicine, The University of Hong Kong, Hong Kong SAR, China; 5 State Key Laboratory of Brain and Cognitive Sciences, The University of Hong Kong, Hong Kong SAR, China; 6 School of Social Sciences, Nottingham Trent University, Nottingham, UK; 7 NTU Psychology, School of Social Sciences, Nottingham Trent University, Nottingham, NG1 6AA UK

**Keywords:** Physical activity, Screen exposure time, Suspected developmental coordination disorder, Joint association, Preschoolers

## Abstract

**Background:**

Developmental coordination disorder (DCD) is a common neurodevelopmental condition in preschoolers. Physical activity (PA) and screen exposure are two highly prevalent and modifiable daily behaviors in early childhood, but evidence regarding their joint association with DCD is limited. This study aimed to investigate the joint associations of PA and screen exposure time (SET) with subsequent suspected DCD in Chinese preschoolers.

**Methods:**

This study used data from a nationally representative, stratified cluster survey covering 551 cities across China. Motor coordination was assessed by the Little Developmental Coordination Disorder Questionnaire (LDCDQ), and suspected DCD was defined according to age- and sex-specific cut-off values. Parent-reported data was used to calculate total daily physical activity (TDPA), moderate-to-vigorous physical activity (MVPA), and daily SET over the previous 12 months. The interactions between PA and SET were examined on both additive and multiplicative scale. Multilevel logistic regression models were employed to explore the association of PA and SET with suspected DCD.

**Results:**

The study included 129,278 preschoolers, among whom 19,969 (15.4%) were suspected DCD. Compared with children in the normal TDPA & SET < 1 h group, the group of low TDPA & SET > 4 h exhibited highest odds of subsequent suspected DCD (aOR = 2.29, 95% CI: 2.02–2.60) when adjusting for covariates; while compared with the normal MVPA & SET < 1 h, those with normal MVPA & SET > 4 h exhibited highest odds of suspected DCD (aOR = 1.96, 95% CI: 1.78–2.15). Analysis of interaction on additive scale (RERI = -0.22, 95% CI: -0.31 to -0.13) and multiplicative scale (aOR = 0.87, 95% CI: 0.80–0.94) indicated that a significantly negative interaction was observed between low MVPA and SET at 1–4 h/day using normal MVPA & SET < 1 h as the reference. Stratified analyses revealed that: (1) low TDPA was a risk factor for suspected DCD at SET ≤ 4 h, whereas low MVPA appeared protective; and (2) across all PA strata, SET ≥ 1 h was significantly associated with the risk of suspected DCD.

**Conclusions:**

Insufficient TDPA, higher MVPA, and excessive SET were associated with a higher risk of subsequent suspected DCD among Chinese preschoolers. A protective interaction was observed between low MVPA and moderate screen exposure (1–4 h/day).

**Supplementary Information:**

The online version contains supplementary material available at https://doi.org/10.1186/s12966-026-01943-8.

## Background

Developmental coordination disorder (DCD) is a common neurodevelopmental condition characterized by persistent impairments in motor coordination. It affects approximately 5–6% of children worldwide and represents a growing public health concern due to its substantial functional and psychosocial consequences [1]. Children with DCD are at increased risk of a broad range of adverse physical and mental health outcomes, including obesity, reduced cardiorespiratory fitness, as well as heightened anxiety, depression, and social withdrawal [2–4]. The pathogenesis of DCD is multifactorial and incompletely understood, with genetic predispositions, preterm birth, family socioeconomic status, maternal lifestyle during pregnancy, and children’s daily behaviours identified as potential contributing factors [5–7]. Among these, modifiable behavioural factors in early childhood are particularly attractive targets for prevention and early intervention [8].

Physical activity (PA) is a fundamental component of healthy child development and plays a critical role in the acquisition and refinement of motor skills [9, 10]. A review demonstrated that most physical activity-based interventions can improve motor proficiency and functional performance in children and adolescents with DCD, and that longer intervention duration was generally associated with greater benefits [11]. Apart from the duration of PA, exercise intensity may also be relevant for improving health in children with DCD [12]. Evidence from a systematic review indicated that high-intensity interval training confers significant benefits for mental health, physical fitness, and cardiometabolic outcomes in children and adolescents with special educational needs, including DCD [13]. Collectively, existing studies have established PA as an effective rehabilitative strategy for children with DCD; however, whether habitual PA patterns in early life are associated with the population-level occurrence of suspected DCD remains largely unexplored.

In parallel, screen exposure has become a dominant daily behaviour among preschool children, particularly in China, alongside the rapid expansion of mobile digital technologies [14, 15]. Our previous national study demonstrated that excessive screen exposure—especially before sleep—was associated with poorer motor competence and increased risk of suspected DCD [16]. From a neurodevelopmental perspective, DCD involves deficits in motor planning and visuomotor integration, and excessive screen exposure may disturb the maturation of visual–sensorimotor pathways, potentially exacerbating the dissociation between visual perception and motor execution [16, 17]. Importantly, contemporary preschool environments are characterised by the coexistence of screen exposure and physical activity within the same daily behavioural repertoire, rather than the presence of either behaviour in isolation [18, 19].

Despite this behavioural reality, how PA and screen exposure jointly associated with the risk of DCD remains poorly understood. In particular, it is unknown whether physical activity modifies the association between screen exposure and DCD, whether excessive screen exposure dominates or attenuates potential protective associations of PA, and whether specific combinations of these may be related to disproportionate risk. To address these gaps, we used data from a large nationally representative survey to examine the joint associations, interaction patterns, and independent roles of PA and screen exposure time (SET) on suspected DCD among Chinese preschool children.

## Methods

### Study design and setting

The present study was part of a large national retrospective cohort study in China to explore neurobehavioral development in Chinese children. A stratified cluster sampling design was implemented to ensure that the participants included in the current study were representative of the children in China. The sampling framework was constructed using stratification criteria derived from the 2018–2019 National Census, including geographic region, age, sex, and socioeconomic status (SES). The sampling procedure included several stages, and we initially selected 551 cities in China as the sampling locations. Subsequently, the government-supported maternity and children’s health center in each city was selected to invite their local kindergartens to participate in the study. Finally, the class teachers were responsible for disseminating the notification to parents to complete the online questionnaire; to facilitate this process and ensure informed consent, contact information of the research team was included for any questions. A data validation protocol was enforced within the online questionnaire system, including pop-up instructions, error messages, links to further information, and setting conditions to ensure participants could not skip questions. A data coordination center was established to oversee all aspects of database management, website maintenance, and inter-institutional coordination, ensuring standardized procedures across all sites.

### Participants

**Fig. 1:**
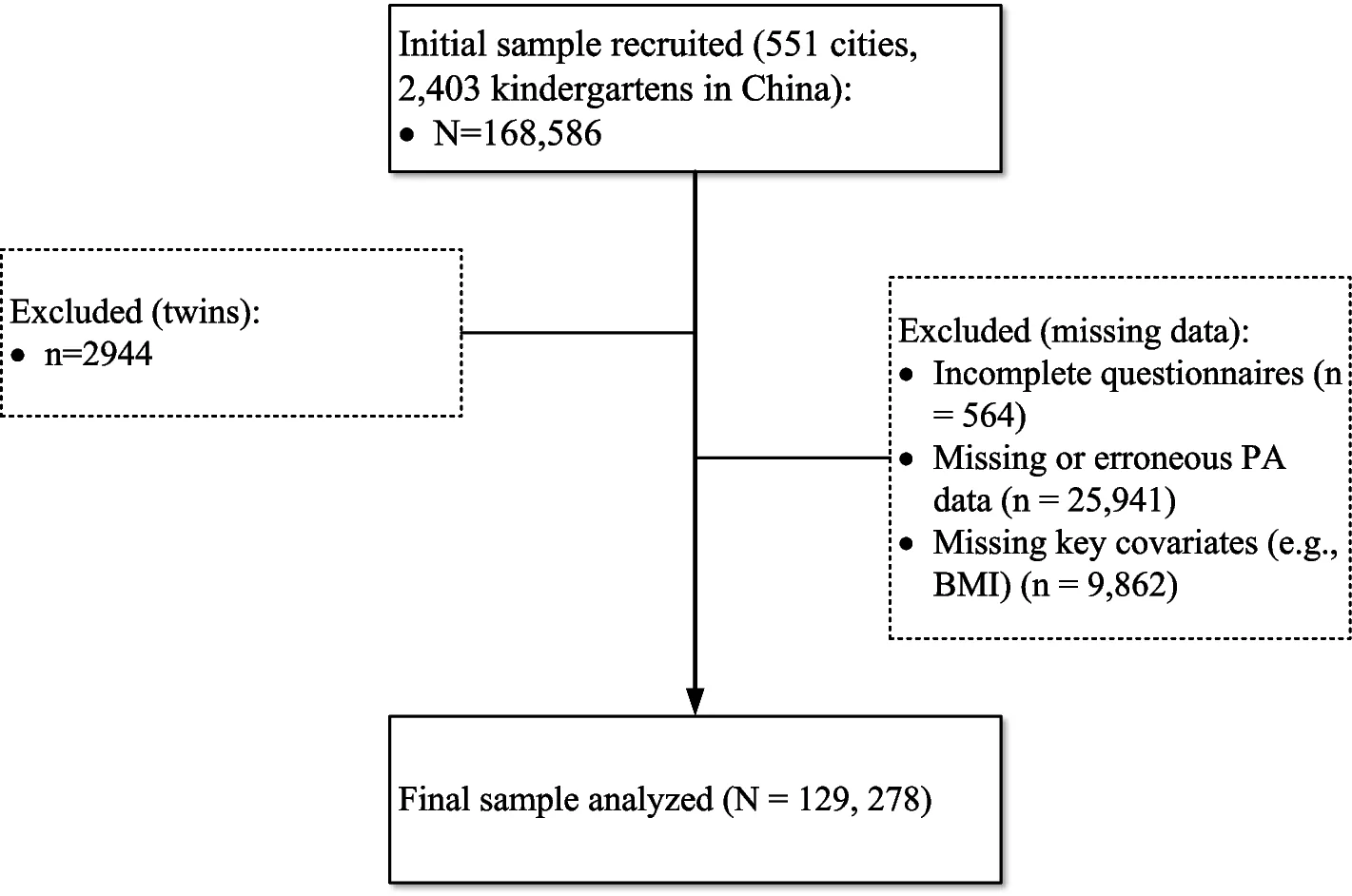
The sample selection process of this study

From April 2018 to December 2019, a total of 168,586 preschoolers (aged 3–5 years) were recruited from 2,403 mainstream kindergartens in 551 cities of China. In the survey, we used the following criteria to include participants: (1) attended a mainstream kindergarten or nursery, (2) children were typically developing without severe visual, hearing, intellectual, or other developmental impairments requiring special education, and (3) parents or guardians could complete an online questionnaire. Conversely, we excluded specific participants based on the following criteria: (1) the children were from twins families to eliminate intra-familial clustering effects and minimize confounding, and (2) had missing data on any of the key variables analyzed in the study. Eventually, there were 129,278 children being selected for analysis in this study (Fig. 1). Additionally, sensitivity analysis comparing included and excluded samples showed highly uniform demographic distributions with difference under 5%, confirming the absence of substantial attrition bias (Table S1).

### Exposure assessment

Physical activity levels were assessed via a parent-reported questionnaire capturing both duration and intensity in the past 12 months, and parents reported their children's activities on typical weekdays and weekends. Total daily physical activity (TDPA) was measured by the question "How long the cumulative time did your child spend on total physical activities per day? ", and similarly, moderate-to-vigorous physical activity (MVPA) was measured as "How long the cumulative time did your child spend on moderate-to-vigorous activity per day? " The questionnaire (Table S2) was developed based on the World Health Organization (WHO) Guidelines on physical activity for children under 5 years of age, and has been utilized in several large-scale pediatric epidemiological studies in China [20–22]. TDPA and MVPA were calculated using a weighted average: (5/7 × weekday activity) + (2/7 × weekend activity). According to the WHO guidelines [22], which recommend at least 180 min of total daily activity and 60 min of MVPA, children were categorized into two groups: those with low TDPA (< 180 min/day) or low MVPA (< 60 min/day), and those with normal levels meeting or exceeding these thresholds.

Parents were also asked to recall the total average duration their child watched TV, used the computer, and used video games. Participants rated their screen time according to the following three questions: ‘How much time during a week day does your child usually watch TV, smartphones, playing computer games or other video games?’ and ‘How much time during a weekend day does your child usually watch TV, smartphones, playing computer games or other video games?’, and ‘How much time does your child usually watch TV, smartphones, playing computer games or other video games before sleep?’ The questions addressed weekdays and weekend days combined for analysis (calculated as: screen time = 5/7 × screen time during weekdays + 2/7 × screen time on weekends). Participants were considered to meet screen time recommendations when they spent less than 1 h per day according to the WHO recommendations that children under age five should spend one hour or less on digital devices [22]. For the analysis, children's daily SET was categorized into three groups: SET < 1 h, SET = 1–4 h, and SET > 4 h.

### Outcomes

Suspected DCD was evaluated using the Little Developmental Coordination Disorder Questionnaire (LDCDQ), a parent-report instrument designed to identify motor coordination difficulties in children aged 3 to 5 years [23, 24]. The questionnaire comprised 15 items categorized into three domains: control during movement, fine motor, and general coordination. Each domain contained five items, and for each item, parents or guardians rated their children's performance relative to same-aged and same-sex peers on a 5-point Likert scale, ranging from 1 ("not at all relevant to my child") to 5 ("extremely relevant to my child"). Domain scores were calculated by summing the five items within each, yielding a maximum of 25 points. These were then totaled for a global score out of 75, where a higher score reflected greater motor proficiency. A previous study reported that the Chinese version of the LDCDQ had good reliability and validity [25]. Based on previous recommendations [24, 26], we used the age- and sex-specific norms of the LDCDQ and cut-off scores were provided based on a national sample in China: children were classified as having suspected DCD if their LDCDQ total score fell at or below the 15th percentile, and those scoring above this threshold were deemed probably not DCD.

### Confounders

Potential confounders were identified based on the established associations with child development and movement behaviors in previous literature [16, 17]. We included the child, family and maternal health characteristics as potential confounders when exploring the association among PA, SET and suspected DCD (Table 1). Most of these variables were dichotomized into ‘yes’ or ‘no’; body mass index (BMI) was an indicator of obesity based on height and weight (BMI = weight(kg)/height(m)^^2^). Family structures were classified into three types: three-generation (or more) family, nuclear family, and single-parent family. The ‘three-generation (or more) family’ referred to the child living with his/her parents and grandparents, which was a traditional family structure in China; ‘nuclear family’ referred to the child living only with his/her parents; and ‘single mother or father’ meant the child lived with one of his/her parents. We divided maternal age into three age bands: ‘ < 30’,’30–34’, and ‘ > 34’ years. Maternal complications of pregnancy and delivery were defined according to the International Classification of Diseases-Revision 10 (ICD-10). The classification was defined as having one of the following maternal complications during pregnancy: vaginal bleeding during pregnancy, risk of miscarriage, use of antibiotics, use of fertility drugs, intrauterine distress, or fetal asphyxia.

**Table 1:** The child, family characteristics and maternal health in the study population (*n* = 129,278)

Variables	Groups	Total	Suspected DCD		*P-value*
			Yes	No	
Children’s age	3	43,661 (33.8)	6712 (33.6)	36,949 (33.8)	0.001
	4	48,531 (37.5)	7716 (38.6)	40,815 (37.3)	
	5	37,086 (28.7)	5541 (27.7)	31,545 (28.9)	
Children’s Gender	Male	67,780 (52.4)	10,107 (50.6)	57,673 (52.8)	< 0.001
	Female	61,498 (47.6)	9862 (49.4)	51,636 (47.2)	
Children’s BMI^a^	-	15.602 ± 1.617	15.617 ± 1.637	15.599 ± 1.613	0.161
Right handedness	No	8748 (6.8)	1633 (8.2)	7115 (6.5)	< 0.001
	Yes	120,530 (93.2)	18,366 (91.8)	102,194 (93.5)	
Eyesight	Normal	120,180 (93.0)	18,257 (91.4)	101,923 (93.2)	< 0.001
	Abnormal	9098 (7.0)	1712 (8.6)	7386 (6.8)	
Birth weight	< 2500 g	4893 (3.8)	962 (4.8)	3931 (3.6)	< 0.001
	≥ 2500 g	124,385 (96.2)	19,007 (95.2)	105,378 (96.4)	
Preterm birth	No	103,344 (79.9)	15,231 (76.3)	88,113 (80.6)	< 0.001
	Yes	25,934 (20.1)	4738 (23.7)	21,196 (19.4)	
Delivery Mode	Vaginal delivery	67,160 (52.0)	10,483 (52.5)	56,677 (51.8)	0.003
	Caesarean section	62,118 (48.1)	9486 (47.5)	52,632 (48.2)	
NICU admission	No	115,926 (89.7)	17,686 (88.6)	98,240 (89.9)	< 0.001
	Yes	13,352 (10.3)	2283 (11.4)	11,069 (10.1)	
Other developmental disorders^b^	No	128,302 (99.2)	19,617 (98.2)	108,685 (99.4)	< 0.001
	Yes	976 (0.8)	352 (1.8)	624 (0.6)	
Psychiatric medication	No	128,242 (99.2)	19,776 (99.0)	108,466 (99.2)	0.004
	Yes	1036 (0.8)	193 (1.0)	843 (0.8)	
Higher education of mother	No	58,862 (45.5)	12,929 (64.7)	45,933 (42.0)	< 0.001
	Yes	70,416 (54.5)	7040 (35.3)	63,376 (58.0)	
Higher education of father	No	60,013 (46.4)	12,702 (63.6)	47,311 (43.3)	< 0.001
	Yes	69,265 (53.6)	7267 (36.4)	61,998 (56.7)	
Mother’s occupation	Employed	107,606 (83.2)	15,625 (78.2)	91,981 (84.1)	< 0.001
	Unemployed	21,672 (16.8)	4344 (21.8)	17,328 (15.9)	
Father’s occupation	Employed	125,431 (97.0)	18,986 (95.1)	109,445 (97.4)	< 0.001
	Unemployed	3847 (3.0)	983 (4.9)	2864 (2.6)	
Family annual per-capita income (RMB)^c^	Below	32,851 (25.4)	6168 (30.9)	26,683 (24.4)	< 0.001
	Above or equal to	96,427 (74.6)	13,801 (69.1)	82,626 (75.6)	
Family structure	Single families	3200 (2.5)	632 (3.2)	2568 (2.3)	< 0.001
	Nuclear families	79,952 (61.8)	13,071 (65.5)	66,881 (61.2)	
	Extended families	46,126 (35.7)	6266 (32.4)	39,860 (36.5)	
The number of children in the family	One	58,019 (44.9)	9700 (48.6)	48,319 (44.2)	< 0.001
	Two or more	71,259 (55.1)	10,269 (51.4)	60,990 (55.8)	
Maternal age at delivery	< 30	95,915 (74.2)	14,922 (74.7)	80,993 (74.1)	< 0.001
	30–34	25,007 (19.3)	3609 (18.1)	21,398 (19.6)	
	≥ 35	8356 (6.5)	1438 (7.2)	6918 (6.3)	
Smoking or passive smoking during pregnancy	No	93,487 (72.3)	13,925 (69.7)	79,562 (72.8)	< 0.001
	Yes	35,791 (27.7)	6044 (30.3)	29,747 (27.2)	
Maternal complications during pregnancy^d^	No	123,032 (95.2)	19,041 (95.4)	103,991 (95.1)	0.187
	Yes	6246 (4.8)	928 (4.6)	5318 (4.9)	
TDPA	Normal	65,008 (50.3)	9405 (47.1)	55,603 (50.9)	< 0.001
	Low	64,270 (49.7)	10,564 (52.9)	53,706 (49.1)	
MVPA	Normal	88,896 (68.8)	14,786 (74.0)	74,110 (67.8)	< 0.001
	Low	40,382 (31.2)	5183 (26.0)	35,119 (32.2)	
SET	SET < 1 h	42,550 (32.9)	4576 (22.9)	37,974 (34.7)	< 0.001
	SET = 1–4 h	82,233 (63.6)	14,283 (71.5)	67,950 (62.2)	
	SET > 4 h	4495 (3.8)	1110 (5.6)	3385 (3.1)	
ALL	-	129,278 (100)	19,969 (15.4)	109,309 (84.6)	

Categorical and continuous variables were described using N (%) and Mean ± SD respectively

^a^The unit for BMI is kg/m^2^ and the independent samples t-test is used for intergroup comparison

^b^Other developmental disorders included autism spectrum disorder, attention deficit/hyperactivity disorder, learning disorders and so on

^c^The national average family per-capita income of the year before the survey time

^d^Having one of the following maternal complications during pregnancy including vaginal bleeding during pregnancy, at risk of miscarriage, use of antibiotics, use of fertility drugs, intrauterine distress, fetal asphyxia

### Statistical analysis

The description was made by use of the mean ± SD (standard deviation) for continuous variables, and number (N) and percentage (%) for categorical variables. Univariate comparisons between groups were performed using independent t-tests for continuous variables and Chi-square tests for categorical variables. Besides, Chi-square tests were employed to assess the prevalence differences of suspected DCD under various combinations of SET and PA.

Given the hierarchical structure of the data, with children nested within kindergartens and kindergartens nested within cities, multilevel logistic regression models were employed to account for potential clustering at both the kindergarten and city levels. Specifically, these models were applied to examine the joint association of PA and SET on suspected DCD, while adjusting for all childhood characteristics, family factors, and maternal health variables during pregnancy as fixed effects. The interaction between PA and SET was examined on both the additive scale and the multiplicative scale, as they capture distinct aspects of effect modification [27]. Multiplicative interaction reflects relative risk heterogeneity and is commonly used for statistical inference, while additive interaction assesses deviation from the sum of their potential risks. From a public health perspective, the additive scale is particularly prioritized as it better identifies specific subpopulations that would derive the greatest absolute benefit from targeted exposure. Additive interactions were assessed by estimating the relative risk due to the interaction (RERI) which represented the additional risk associated with having both risk factors above the sum of the individual risk factors [27]. Multiplicative interactions were examined in a separate model including individual PA and SET variables and an interaction term.

Following the recommendations for presenting interaction analyses (Knol & VanderWeele, 2012), we conducted stratified analyses to examine the independent role of exposures (PA or SET) on suspected DCD. The study population was stratified into subgroups based on the levels of PA and SET, where the reference group was re-defined within each stratum (e.g., normal PA as the reference within each SET category). Within each subgroup, separate multilevel logistic regression models were employed to estimate the odds size of the exposure factor along with its 95% confidence interval (CI). This analysis aimed to quantify the observed interaction, provide subgroup-specific association estimates, facilitate public health interpretation, and serve as a robustness check for the interaction model.

In these models adjusting for confounders, all the variance inflation factor (VIF) values were well below the conventional threshold of 5 (the maximum VIF was 1.81), indicating that multicollinearity did not significantly bias our estimation. A statistical significance level was set at *P-value* < 0.05 (two tailed). All analyses were performed with the Stata/MP version 17.0 (StataCorp, College Station, TX, USA).

## Results

### Characteristics of the participants

Table 1 presents the detailed characteristics of the study population. A total of 129,278 preschool children were included in the analysis, among whom 19,969 (15.4%) were classified as having suspected DCD. Compared with children without suspected DCD, those with suspected DCD were less likely to have parents with higher educational attainment (mothers: 35.3% vs. 64.7%; fathers: 36.4% vs. 63.6%). Children with suspected DCD were also less frequently drawn from families with lower annual per-capita income (30.9% vs. 69.1%). In addition, a higher proportion of children with suspected DCD had experienced preterm birth (23.7%), neonatal intensive care unit (NICU) admission (11.4%), and maternal active or passive smoking during pregnancy (30.3%). In terms of PA, 49.7% and 31.2% of the total sample showed low level for TDPA and MVPA, respectively. The proportion of SET lasting 1–4 h and > 4 h per day was 63.6% and 3.8%, respectively. Univariate analysis indicated that the distribution of children’s BMI (*P* = 0.161) and maternal complications during pregnancy (*P* = 0.187) showed no significant differences between the suspected DCD and non-suspected DCD groups. In contrast, all other variables were found to be statistically significant (all *P* < 0.05).

### Prevalence of suspected DCD under different PA and SET combinations

**Fig. 2:**
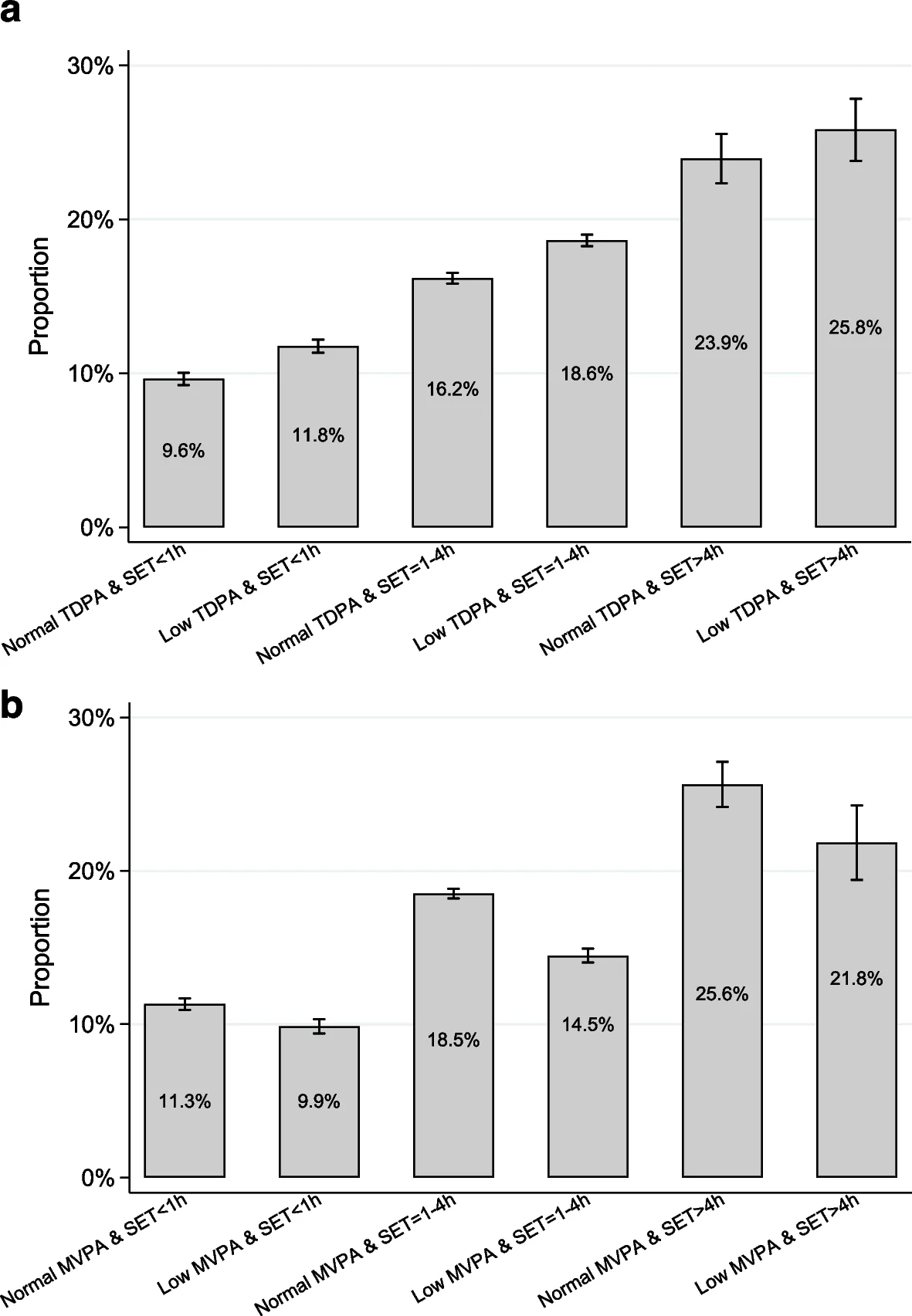
Prevalence of suspected DCD under different TDPA/MVPA and SET combinations. **a** Prevalence of suspected DCD under TDPA and SET combinations. **b** Prevalence of suspected DCD under MVPA and SET combinations. Notes: TDPA = Total daily physical activity, SET = Screen exposure time, MVPA = Moderate-to-vigorous physical activity

As illustrated in Fig. 2, across the six combinations of total daily physical activity (TDPA) and screen exposure time (SET), the prevalence of suspected DCD was lowest among children with normal TDPA and SET < 1 h (9.6%). A graded pattern was observed, with the prevalence of suspected DCD increasing progressively as TDPA decreased and SET increased, reaching 25.8% among children with low TDPA and SET > 4 h. The distribution of suspected DCD differed significantly across TDPA–SET groups (Chi-square tests: χ^2^ = 1378.6, *P* < 0.001). In contrast, a distinct pattern was observed for combinations of MVPA and SET. The lowest prevalence of suspected DCD was observed in children with low MVPA and SET < 1 h (9.9%), whereas the highest prevalence was observed in those with normal MVPA and SET > 4 h (25.6%). Differences in the prevalence of suspected DCD across MVPA–SET combinations were also statistically significant (Chi-square tests: χ^2^ = 1399.1, *P* < 0.001).

### Joint association of PA and SET with suspected DCD

As shown in Table 2, multilevel logistic regression models showed that using children with normal TDPA and SET < 1 h as the reference group, those with low TDPA and SET < 1 h had higher odds of suspected DCD after adjustment for maternal and child characteristics (adjusted odds ratio [aOR] = 1.24, 95% CI: 1.16–1.33). The highest odds were observed among children with low TDPA and SET > 4 h (aOR = 2.29, 95% CI: 2.02–2.60) compared with the children with normal TDPA and SET < 1 h. Using children with normal MVPA and SET < 1 h as the reference group, children with low MVPA and SET < 1 h showed slightly lower odds of suspected DCD (aOR = 0.90, 95% CI: 0.84–0.96), whereas those with normal MVPA and SET > 4 h exhibited the highest odds (aOR = 1.96, 95% CI: 1.78–2.15).

**Table 2:** The joint association of PA and SET with suspected DCD in preschoolers (*n* = 129,278)

Categories	Adjusted OR^a^ (95% CI)	*P-value*	Additive interaction (RERI, 95%CI)	Multiplicative interaction (OR, 95%CI)
Joint association of TDPA & SET				
Normal TDPA & SET < 1 h	Ref			
Low TDPA & SET < 1 h	1.241 (1.162, 1.325)	< 0.001		
Normal TDPA & SET = 1–4 h	1.480 (1.397, 1.567)	< 0.001		
Low TDPA & SET = 1–4 h	1.738 (1.642, 1.840)	< 0.001	0.017 (−0.078, 0.113)	0.946 (0.877, 1.021)
Normal TDPA & SET > 4 h	2.140 (1.919, 2.385)	< 0.001		
Low TDPA & SET > 4 h	2.290 (2.017, 2.601)	< 0.001	−0.091 (−0.435, 0.253)	0.862 (0.732, 1.016)
Joint association of MVPA & SET				
Normal MVPA & SET < 1 h	Ref			
Low MVPA & SET < 1 h	0.899 (0.840, 0.963)	0.002		
Normal MVPA & SET = 1–4 h	1.463 (1.396, 1.533)	< 0.001		
Low MVPA & SET = 1–4 h	1.144 (1.081, 1.211)	< 0.001	**−0.218** **(−0.308, −0.130)**	**0.869** **(0.801, 0.943)**
Normal MVPA & SET > 4 h	1.956 (1.780, 2.150)	< 0.001		
Low MVPA & SET > 4 h	1.665 (1.418, 1.955)	< 0.001	−0.190 (−0.504, 0.123)	0.947 (0.784, 1.143)

Bold typeface is used to highlight statistically significant results

*TDPA* Total daily physical activity, *SET* Screen exposure time, *MVPA* Moderate-to-vigorous physical activity

^a^Multilevel logistic regression models were used to address clustering association, and the full set of covariates (childhood, family, and maternal health characteristics) have been adjusted in the models

Formal tests of interaction indicated no significant interaction between TDPA and SET on neither the additive nor multiplicative scale (*P* > 0.05). In contrast, a significant antagonistic interaction was observed between low MVPA and SET of 1–4 h per day, with normal MVPA and SET < 1 h as the reference. On the additive scale, the relative excess risk due to interaction (RERI) was − 0.22 (95% CI: − 0.31 to − 0.13), and on the multiplicative scale, the interaction term odds ratio was 0.87 (95% CI: 0.80–0.94). This antagonistic interaction indicated that the combined risk associated with both low MVPA and moderate screen exposure was lower than would be expected if their individual risks were simply added together.

### Stratified analysis on the association of PA and SET with suspected DCD

#### The association of PA with suspected DCD when stratified by SET

As shown in Table 3, multilevel logistic regression models showed that among children with SET < 1 h per day, low TDPA was associated with higher odds of suspected DCD after adjustment for covariates (aOR = 1.27, 95% CI: 1.19–1.36) compared with the normal TDPA group within this stratum. In contrast, low MVPA was associated with lower odds of suspected DCD (aOR = 0.86, 95% CI: 0.80–0.92) compared with the normal MVPA group within this stratum. A similar pattern was observed among children with SET of 1–4 h per day. However, within the SET > 4 h/day stratum—using children with normal TDPA as the reference group—low TDPA was not significantly associated with increased odds of suspected DCD (aOR = 1.08, 95% CI: 0.93–1.26).

**Table 3:** The association between PA and suspected DCD when stratified by SET using multilevel logistic regression models

**Categories**	Crude OR (95% CI)	*P-value*	Adjusted OR^a^ (95% CI)	*P-value*
In children with SET < 1 h				
TDPA Normal	Ref		Ref	
Low	1.296 (1.216, 1.381)	< 0.001	1.272 (1.190, 1.360)	< 0.001
MVPA Normal	Ref		Ref	
Low	0.836 (0.783, 0.893)	< 0.001	0.859 (0.802, 0.921)	< 0.001
In children with SET = 1–4 h				
TDPA Normal	Ref		Ref	
Low	1.227 (1.183, 1.274)	< 0.001	1.205 (1.159, 1.253)	< 0.001
MVPA Normal	Ref		Ref	
Low	0.734 (0.704, 0.767)	< 0.001	0.765 (0.731, 0.801)	< 0.001
In children with SET > 4 h				
TDPA Normal	Ref		Ref	
Low	1.119 (0.974, 1.286)	0.113	1.080 (0.928, 1.256)	0.321
MVPA Normal	Ref		Ref	
Low	0.808 (0.687, 0.952)	0.011	0.830 (0.695, 0.991)	0.040

*TDPA* Total daily physical activity, *SET* Screen exposure time, *MVPA* Moderate-to-vigorous physical activity

^a^Multilevel logistic regression models were used to address clustering association, and the full set of covariates (childhood, family, and maternal health characteristics) have been adjusted in the models

#### The association of SET with suspected DCD when stratified by PA

As shown in Table 4, multilevel logistic regression models were used to evaluate the robustness of SET risks, considering children with SET < 1 h/day served as the internal reference group. The results showed a consistent and graded increase in the odds of suspected DCD with rising SET levels, regardless of whether children had normal or low levels of TDPA or MVPA (*P* < 0.001). Specifically, using children with SET < 1 h per day as the reference group, those with SET of 1–4 h per day exhibited at least a 29.7% increase in the odds of suspected DCD (lowest aOR = 1.297, 95% CI: 1.21–1.39). While this association for children with SET > 4 h per day was no less than an 89% increase in the odds of suspected DCD (lowest aOR = 1.89, 95% CI: 1.67–2.14) relative to the reference group of SET < 1 h/day. These findings confirmed that excessive screen exposure was a stable risk factor that persists independent of physical activity levels.

**Table 4:** The association between SET and suspected DCD when stratified by PA using multilevel logistic regression models

**Categories**	Crude OR (95% CI)	*P-value*	Adjusted OR^a^ (95% CI)	*P-value*
In children with normal TDPA				
SET < 1 h	Ref		Ref	
SET = 1–4 h	1.751 (1.659, 1.849)	< 0.001	1.451 (1.369, 1.537)	< 0.001
SET > 4 h	2.829 (2.556, 3.132)	< 0.001	2.077 (1.861, 2.318)	< 0.001
In children with low TDPA				
SET < 1 h	Ref		Ref	
SET = 1–4 h	1.692 (1.611, 1.775)	< 0.001	1.426 (1.353, 1.503)	< 0.001
SET > 4 h	2.567 (2.289, 2.879)	< 0.001	1.891 (1.667, 2.144)	< 0.001
In children with normal MVPA				
SET < 1 h	Ref		Ref	
SET = 1–4 h	1.734 (1.659, 1.812)	< 0.001	1.450 (1.383, 1.520)	< 0.001
SET > 4 h	2.607 (2.389, 2.846)	< 0.001	1.934 (1.759, 2.127)	< 0.001
In children with low MVPA				
SET < 1 h	Ref		Ref	
SET = 1–4 h	1.524 (1.429, 1.625)	< 0.001	1.297 (1.210, 1.390)	< 0.001
SET > 4 h	2.528 (2.169, 2.945)	< 0.001	1.910 (1.617, 2.255)	< 0.001

*TDPA* Total daily physical activity, *SET* Screen exposure time, *MVPA* Moderate-to-vigorous physical activity

^a^Multilevel logistic regression models were used to address clustering association, and the full set of covariates (childhood, family, and maternal health characteristics) have been adjusted in the models

## Discussion

Using data from a large, nationally representative survey of Chinese preschoolers, we examined the joint associations of PA and SET with suspected DCD. We identified a clear and graded behavioral pattern, whereby insufficient TDPA combined with increasing screen exposure was associated with progressively higher odds of suspected DCD, with screen exposure emerging as a dominant and threshold-dependent risk factor. Notably, excessive screen exposure appeared to override the potential protective associations of PA, while specific PA–SET behavioural combinations exhibited antagonistic interactions, underscoring the importance of considering the overall composition of children’s daily movement behaviors rather than isolated activity domains.

We observed a clear behavioural gradient, whereby decreasing TDPA combined with increasing screen exposure was associated with a progressively elevated risk of suspected DCD. Children characterized by the most adverse behavioral profile—low TDPA and high SET (> 4 h/day)—exhibited both the highest prevalence (25.8%) and the greatest odds of suspected DCD (aOR = 2.29), indicating a non-additive joint association. These findings extended prior studies that have predominantly examined PA or screen exposure in isolation and suggested that the co-occurrence of insufficient movement and excessive screen-based sedentary behavior constituted a critical behavioral phenotype for DCD risk in early childhood [11, 28]. Several neurodevelopmental pathways may underlie these associations. Excessive screen exposure has been linked to alterations in dorsal striatal connectivity and disruptions in attentional and executive control networks [29, 30], processes closely coupled with motor planning and coordination. Concurrently, prolonged screen use increases sedentary time while displacing free play and outdoor activities essential for the acquisition and refinement of fundamental motor skills [21, 31, 32]. Reduced opportunities for diverse sensorimotor experiences may compromise muscle strength, coordination, and motor learning, thereby increasing susceptibility to DCD. From a public health perspective, these findings suggest that a substantial proportion of suspected DCD burden in preschoolers may be attributable to modifiable behavioral factors, highlighting opportunities for early identification and intervention [33].

Notably, elevated odds of suspected DCD were observed among children with prolonged screen exposure (> 4 h/day) even when recommended levels of MVPA were met. This finding indicates that gross motor activity alone may be insufficient to counterbalance the neurodevelopmental risks associated with excessive digital screen use [34, 35]. Prolonged screen use can interfere with developmentally necessary motor repetition, visuomotor integration, and proprioceptive feedback, thereby impeding coordination even in children engaging in recommended MVPA levels [36–38]. Together, these results challenge the assumption that meeting MVPA guidelines necessarily confers protection against motor developmental risk in the context of high screen exposure.

Interaction analyses further revealed a significant antagonistic interaction between low MVPA and moderate SET (1–4 h/day) on both additive and multiplicative scales, corresponding to a comparatively reduced burden of suspected DCD under this specific behavioral combination. From a behavioral perspective, children’s daily movement behaviours constitute a dynamic, multidimensional system in which different categories of activities may interact through substitution and compensatory value [39, 40]. Low MVPA in this context should not be equated with physical inactivity; instead, children with lower MVPA may accumulate substantial light-intensity physical activity alongside moderate screen-based observational or attentional behaviours [41]. Such behavioural compositions may still support adequate motor experience, visuomotor integration, and fine motor engagement during sensitive developmental windows [42–44], offering a plausible explanation for the observed antagonistic interaction.

Interestingly, stratified analyses by SET further indicated that low TDPA was associated with increased risk, whereas low MVPA appeared protective within one hour. This divergence likely reflects differences in the constructs captured by these measures. MVPA primarily reflects gross motor activity, whereas the discrepancy between TDPA and MVPA may represent engagement in light-intensity and fine motor activities—such as drawing, manipulation, and tool use—which are critical for motor coordination development in preschoolers [3, 45, 46]. These findings suggest that interventions focusing exclusively on increasing MVPA may be insufficient, and that promoting a balanced composition of daily movement behaviors, including fine motor skill–enriching activities, may be particularly important.

Importantly, when SET exceeded 4 h/day, associations between TDPA and suspected DCD were no longer apparent, suggesting that excessive screen exposure may override or neutralize the potential protective factor of physical activity in preschool children. Once screen use surpasses a critical threshold, the adverse neurobehavioral consequences of prolonged sedentary exposure may outweigh the developmental benefits from physical activity, resulting in null or attenuated associations [47, 48]. This threshold association is consistent with the competitive hypothesis, whereby excessive screen time displaces developmentally essential physical, social, and play-based experiences [49, 50]. Accordingly, early behavioral interventions should prioritize reducing excessive screen exposure as a primary target, alongside promoting active play as a replacement rather than a simple additive behavior [51].

Additional stratified analyses by PA demonstrated that any behavioral pattern involving SET ≥ 1 h/day was consistently associated with elevated odds of suspected DCD. Screen exposure of 1–4 h/day was associated with at least about 30% increase in risk, while exposure exceeding 4 h/day approximately triple the risk. These findings reinforce international movement guidelines advocating minimal screen exposure in early childhood and suggest that interventions focusing solely on increasing PA may be insufficient in the absence of concurrent screen-time reduction [52, 53]. Future research should prioritize elucidating the mechanistic and causal pathways linking screen exposure to DCD, which may inform targeted, evidence-based prevention strategies emphasizing early screen-time regulation.

Several limitations warrant consideration. First, although this study was based on a large, nationally representative sample of preschool children, the retrospective design precludes causal inference regarding the associations between SET, PA, and suspected DCD. Consequently, future randomized controlled trials (RCTs) are needed to further evaluate the potential causal nature of these associations. Second, key exposure variables and covariates were derived from parent-reported questionnaires, which may be subject to recall bias and exposure misclassification, particularly for estimates of screen use and physical activity duration. Third, despite extensive adjustment for child-, family-, and maternal-level characteristics, residual confounding—such as genetic susceptibility and early neurodevelopmental history—cannot be entirely excluded. Future studies incorporating objective behavioral assessments, standardized clinical evaluations, and repeated longitudinal measurements will be essential to enhance measurement precision and strengthen causal inference.

## Conclusions

This study demonstrated that insufficient TDPA, more MVPA, and excessive SET were associated with a higher risk of subsequent suspected DCD among Chinese preschoolers, with screen time ≥ 1 h/day serving as a dominant and graded risk factor largely independent of activity level. When screen exposure exceeded 4 h/day, the associations between physical activity and suspected DCD were attenuated, suggesting that excessive screen use may override the potential developmental benefits of physical activity. Moreover, a significant antagonistic interaction was observed between low MVPA and moderate screen exposure (1–4 h/day), underscoring the complexity of children’s daily movement behaviour compositions. Collectively, these findings highlight the importance of early, family-centered behavioural strategies that jointly address screen use and physical activity to support optimal motor development in preschool children.

## Supplementary Information

Supplementary Material 1.

Supplementary Material 2.

## Data Availability

The datasets generated and analyzed during the current study are not publicly available due to privacy protection and ethical restrictions but are available from the corresponding author on reasonable request.

## Abbreviations

DCD: Developmental coordination disorder
PA: Physical activity
SET: Screen exposure time
SES: Socioeconomic status
TDPA: Total daily physical activity
MVPA: Moderate-to-vigorous physical activity
LDCDQ: Little Developmental Coordination Disorder Questionnaire
BMI: Body mass index
ICD-10: International Classification of Diseases-Revision 10
SD: Standard deviation
RERI: Relative risk due to the interaction
CI: Confidence interval
NICU: Neonatal intensive care unit
aOR: Adjusted odds ratio
