# Supplementary material for: Joint association of physical activity and screen exposure with subsequent suspected developmental coordination disorder in preschool children: a nationwide population-based study
Source: Int J Behav Nutr Phys Act. 2026 Jul 6;23:89. doi: 10.1186/s12966-026-01943-8 (PMC13632386; doi:10.1186/s12966-026-01943-8)
Supplement: Supplementary file 1 [file 12966_2026_1943_MOESM1_ESM.docx]

**Table S1. Comparison of sociodemographic and clinical characteristics between the analytical sample and excluded individuals. (n=168,580)**

| Variables | Groups | Total | Excluded Sample | Included Sample | Absolute Difference (%) | *P-value* |
| --- | --- | --- | --- | --- | --- | --- |
| Children’s age | 3 | 58589 | 14928 (38.0) | 43661 (33.8) | 4.2 | <0.001 |
|  | 4 | 62947 | 14416 (36.7) | 48531 (37.5) | 0.8 |  |
|  | 5 | 47044 | 9958 (25.3) | 37086 (28.7) | 3.4 |  |
| Children’s Gender | Male | 88832 | 21052 (53.6) | 67780 (52.4) | 1.2 | <0.001 |
|  | Female | 79748 | 18250 (46.4) | 61498 (47.6) | 1.2 |  |
| Children’s BMI^a^ | - | 15.61±1.73 | 15.64±2.11 | 15.60±1.62 | 0.04(Mean) | 0.001 |
| Right handedness | No | 11942 | 3194 (8.1) | 8748 (6.8) | 1.3 | <0.001 |
|  | Yes | 156638 | 36108 (91.9) | 120530 (93.2) | 1.3 |  |
| Eyesight | Normal | 155829 | 35649 (90.7) | 120180 (93.0) | 2.3 | <0.001 |
|  | Abnormal | 12751 | 3653 (9.3) | 9098 (7.0) | 2.3 |  |
| Birth weight* | <2500g | 6323 | 1430 (3.7) | 4893 (3.8) | 0.1 | 0.751 |
|  | ≥2500g | 161092 | 36707 (96.3) | 124385 (96.2) | 0.1 |  |
| Preterm birth | No | 136635 | 33291 (84.7) | 103344 (79.9) | 4.8 | <0.001 |
|  | Yes | 31945 | 6011 (15.3) | 25934 (20.1) | 4.8 |  |
| Delivery Mode | Vaginal delivery | 86783 | 19623 (49.9) | 67160 (52.0) | 2.1 | <0.001 |
|  | Caesarean section | 81797 | 19679 (50.1) | 62118 (48.1) | 2.0 |  |
| NICU admission | No | 150988 | 35062 (89.2) | 115926 (89.7) | 0.5 | 0.009 |
|  | Yes | 17592 | 4240 (10.8) | 13352 (10.3) | 0.5 |  |
| Other developmental disorders^b^ | No | 167490 | 39188 (99.7) | 128302 (99.2) | 0.5 | <0.001 |
|  | Yes | 1090 | 114 (0.3) | 976 (0.8) | 0.5 |  |
| Psychiatric medication | No | 167376 | 39134 (99.6) | 128242 (99.2) | 0.4 | <0.001 |
|  | Yes | 1204 | 168 (0.4) | 1036 (0.8) | 0.4 |  |
| Total | - | 168580 | 39302 | 129278 | - | - |

**Notes**: Categorical and continuous variables were described using N (%) and Mean ± SD respectively. ^a^The unit for BMI is kg/m^2^ and the independent samples t-test is used for intergroup comparison. ^b^Other developmental disorders included autism spectrum disorder, attention deficit/hyperactivity disorder, learning disorders and so on. *There are missing values for the ‘Birth weight’ variable in the sample.

A total of 168,586 participants were initially approached, among whom 6 signed the informed consent but did not complete any questionnaire entries, leaving a total of 168,580 valid respondents for this comparative analysis. Due to the exceptionally large sample size, statistical significance (*P* < 0.05) does not reflect substantively meaningful differences, as percentage variations remain under 5.0%.
