# Supplementary material for: Joint association of physical activity and screen exposure with subsequent suspected developmental coordination disorder in preschool children: a nationwide population-based study
Source: Int J Behav Nutr Phys Act. 2026 Jul 6;23:89. doi: 10.1186/s12966-026-01943-8 (PMC13632386; doi:10.1186/s12966-026-01943-8)
Supplement: Supplementary file 2 [file 12966_2026_1943_MOESM2_ESM.docx]

**Table S2. Parent-reported Physical Activity Questionnaire for Preschoolers**

- **Instructions**: Please (as the primary caregiver) recall your child's typical weekly activity patterns over the past year.

| **Items** | **Typical School Day (Kindergarten)** | **Typical Weekend (Sat. & Sun.)** |
| --- | --- | --- |
| **E1: Average daily activity time**  (Total cumulative time of various physical activities throughout the day, such as walking, running, cycling, playing, household chores, and other sports competitions). | _____ Hours _____ Mins | _____ Hours _____ Mins |
| **E2: Time spent in moderate-to-vigorous physical activity (MVPA)**  (Characterized by faster/shortness of breath, facial flushing, feeling tired or exertion, and sweating slightly or heavily). | _____ Hours _____ Mins | _____ Hours _____ Mins |
